# The Bridging Effect of Controlled-Release Glial Cell-Derived Neurotrophic Factor Microcapsules within Nerve Conduits on Rat Facial Nerve Regeneration

**DOI:** 10.1155/2022/8942985

**Published:** 2022-06-21

**Authors:** Siwen Xia, Mingxing Zhang, Meng Li, Xianmin Song, Donghui Chen, Minhui Zhu, Hongliang Zheng, Shicai Chen

**Affiliations:** ^1^Department of Otolaryngology Head & Neck Surgery, The Second Affiliated Hospital and Yuying Children's Hospital, Wenzhou Medical University, 109 Xueyuan West Road, Wenzhou, Zhejiang 325027, China; ^2^Department of Otolaryngology Head & Neck Surgery, Changhai Hospital, Navy Medical University, Shanghai 200433, China; ^3^Hangzhou Renshu Clinic, Xizi international T3B building, 9 Jingtan Road, Hangzhou, Zhejiang 310020, China; ^4^Department of Otorhinolaryngology-Head & Neck Surgery, Jiangsu Province Hospital, The First Affiliated Hospital of Nanjing Medical University, Guangzhou Road #300, Nanjing 210029, China; ^5^Center for Otolaryngology-Head & Neck Surgery of Chinese PLA, Shanghai 200433, China

## Abstract

**Objectives:**

The study is aimed at exploring the effect of the controlled release of the glial-derived neurotrophic factor (GDNF) on nerve regeneration.

**Methods:**

The PLGA/chitosan composite nerve conduit was used to bridge the dissected trunk of the rat facial nerve. GDNF microcapsules were loaded into the nerve conduit. Nine weeks after surgery, the facial nerve zygomatic and buccal branches were labeled with fluorescent indicators. The incorrectly grown facial neurons were reversed and counted. The facial nerve functional recovery was assessed by measuring the maximum evoked potential.

**Results:**

The nerve conduit was filled with different regenerating factors, such as the GDNF, GDNF microcapsules, or saline (control). The number of incorrectly regenerated neurons was lower with the nerve conduits filled with GDNF microcapsules than with those supplied with just the GDNF. However, neither the GDNF nor GDNF microcapsules affected the number of regenerated neurons. The functional recovery of the facial nerve was the best, with the nerve conduit filled with GDNF microcapsules closest to the healthy uncut facial nerve.

**Conclusion:**

The stable slow-release GNDF microcapsule inside the biodegradable nerve conduit can reduce the extent of incorrect growth of the facial nerve neuron when bridging the dissected rat facial nerve trunk. The technique has a good effect on functional nerve recovery.

## 1. Introduction

The facial nerve, the recurrent laryngeal nerve, and other peripheral nerves are more susceptible to injury during trauma, surgery, and tumor growth. Although these nerves can be recovered by surgery, postoperative functional recovery has been disappointing. In particular, synkinesis can occur due to the incorrect regeneration of miscellaneous nerve fibers in the nerve trunk [1]. Neurotrophic factors (NTFs) play an essential role in the nerve regeneration process [2]. Research has confirmed that applying exogenous NTFs combined with nerve conduits to repair nerve injuries can improve curative effects [3, 4]. The glial cell-derived neurotrophic factor (GDNF), secreted by the Schwann cell and retrograde transported to neurons via axons, maintains the survival of neurons and prevents neuron apoptosis. The GDNF is the most potent neurotrophic factor of all GDNFs in the regeneration and protection of motor neurons [5–7].

The ability to achieve guided nerve regeneration has practical clinical significance. When GDNF is used for the entire body, it will induce strong side effects, limiting its clinical application [8]. Researchers have explored using biomedical engineering techniques to realize stable local slow release of the GDNF in the microenvironment to promote nerve regeneration [9]. In this study, we prepared the continuous stable slow release of GDNF microcapsules. The microcapsules were injected into the poly (lactide-co-glycolide) (PLGA) chitosan composite nerve conduit to bridge the dissected rat facial nerve trunk. The effect of the GDNF microcapsules on the guided facial nerve regeneration after nerve injury was explored. This study provides an experimental basis to understand the temporal and special effects of GDNF on motor neuron regeneration during the recovery process.

## 2. Methods

### 2.1. The Preparation of GDNF Solution and GDNF Microcapsule Solution

The optimal effective concentration of GDNF is 200 ng/mL based on our previous experiments and the literature [10]. The GDNF solution was prepared by diluting 20 *μ*L of GDNF stock solution (10 *μ*g/mL) with 670 *μ*L of double-distilled water (total volume 690 *μ*L). The GDNF-PLGA microcapsules were prepared by the school of pharmacy of the Second Military Medical University. After authentication, the loading capacity of each microcapsule is 0.175 *μ*g/mL. The microcapsule can be continuously released for 40 days. The GDNF microcapsule solution was prepared by adding 1.143 mg of GDNF-PLGA microcapsules to double-distilled water (total volume 690 *μ*L).

### 2.2. The Preparation of Collagen-Based Three-Dimensional Scaffold

All preparation procedures were performed at ice-cold temperature. Each 200 *μ*L of type I rat tail collagen (5 mg/mL) was added to three ice-bathed centrifuge tubes. Then, 690 *μ*L of double-distilled water (control), GDNF solution, or GDNF microcapsule solution was added to each tube separately. Subsequently, 0.1 mol/L of NaOH was added to each tube (total volume 12 *μ*L). The procedures must be followed in the order described; otherwise, it will induce collagen coagulation. Each solution was thoroughly mixed and stored at low temperatures for future use [11]. Since type I rat tail collagen can be congealed under room temperature and pH-neutral conditions, all operating procedures should be performed at low temperatures to keep the collagen as liquid for future injection.

### 2.3. The Preparation of Animal Models

Thirty adult male Sprague-Dawley (SD) rats, with body weights of 250 g to 300 g, were purchased from the Shanghai Laboratory Animal Research Center. The study was approved by the Experimental Animal Ethics Committee of Changhai Hospital of The Second Military Medical University (SYXK-2020-0033). All animal experiments followed the Laboratory Animal Management Ordinance regulations and the experimental animal ethics requirement.

SD rats were anesthetized by injecting avertin (250 mg/kg body weight, Sigma-Aldrich). A curved incision was prepared at the spot 0.4 cm below the right bottom side of the auricle cartilage edge. The orbital tear ducts and parotid gland were separated and the forward tract to the deep sternocleidomastoid surface. It was separated upward to the hole in the breast stem and the tendons, and the facial nerve trunk was fully exposed. Under the surgical microscope, the middle part of the nerve trunk was removed. The two ends of the excised nerve truck (approximately 1 mm in length at each end) were introduced into the PLGA/chitosan composite nerve conduit (length 3.5 mm, diameter 1.2 mm), which was prepared by Shanghai Tian Qing Biomaterial Co. Ltd. (Shanghai, China). The epineurium of the nerve trunk close to the heart end was sewed to one end of the nerve conduit with the number 11-0 suture. The epineurium of the telecentric end of the nerve trunk was stitched to the other end of the nerve conduit. Then, the sutures were tightened.

Approximately 5 *μ*L of collagen-based three-dimensional scaffolds containing one of the three components (water, GDNF, and GDNF microcapsule) were injected separately into the nerve conduit with microsyringes. After injections, the sutures connecting the nerve conduit and the excised end of the nerve trunk were tightened and fixed. The animals were injected with penicillin sodium (80,000 units/mouse) to prevent infection three days after surgery. After being fully awake, the animals were placed back in cages. All animals were kept with 12 hours of light cycle change with free access to water.

### 2.4. Facial Nerve Fluorescence Retrograde Labeling and General Morphological Observation on the Bridging Spot

Nine weeks after surgery, the rats were anesthetized with avertin. The zygomatic branch and the buccal branch of the surgical facial lateral nerve were exposed. 1,1′-Dioctadecyl-3,3,3′,3′-tetramethylindocarbocyanine perchlorate (DIL) (Sigma-Aldrich, MO) and fluorescence gold (FG) (Fluorochrome, USA) were injected separately into the zygomatic branch and buccal branch with microsyringes. One week later, the original incision was opened, exposing the right side of the facial nerve trunk. The general morphology of the trunk of the right lateral facial nerve was inspected. Subsequently, rats were perfused with 4% paraformaldehyde and brains were removed for future use.

### 2.5. Retrograde Labeling and Localization of Facial Nerve Neurons and Incorrect Regeneration Analysis

The brain was sectioned to a thickness of 25 *μ*m using a microtome (Leica CM1950). All fluorescent image acquisition was performed with a Leica DL2000 fluorescence microscope. The numbers of motor neurons stained with DIL (red) or FG (blue) were counted for every mouse. The DIL and FG staining pictures were combined into one single image with Photoshop 9.0. A total cell number was obtained from this combined picture. Only neurons with a diameter larger than 10 *μ*m with intact morphology and higher brightness than the background were counted for statistical analysis.

According to the distribution characteristics of the facial nerve nucleus that dominated the zygomatic branch and the buccal branch subnucleus, incorrectly regenerated facial nerves were counted. These incorrectly regenerated facial nerves included double-labeled motor neurons, DIL-stained motor neurons extending from the dorsal nucleus, and FG-stained neurons that intruded into the dorsal nucleus.

The specific calculation method of the extent of incorrect regeneration is as follows:

(1) $$\begin{array}{c} \text{The ratio of zygomatic nerve fiber growing incorrectly to buccal area}=\frac{\text{cell number of DIL stained zygomatic nerve fiber in buccal subnucleus}}{\text{total cell number stained by DIL and FG in buccal subnucleus}},\\ \text{The ratio of buccal nerve fiber growing incorrectly to zygomatic area}=\frac{\text{cell number of FG stained buccal nerve fiber in zygomatic subnucleus}}{\text{total cell number stained by DIL and FG in zygomatic subnucleus}},\\ \text{The total inccorect regeneration ration of facial nerve}=\frac{\begin{array}{c} \text{cell number of DIL stained zygomatic nerve fiber in buccal subnucleus}+\\ \text{cell number of FG stained buccal nerve fiber in zygomatic subnucleus}+\\ \text{double labeled neurons} \end{array}}{\begin{array}{c} \text{total cell number stained by DIL and FG in buccal and zygomatic }\\ \text{subnucleus double labeled neurons in both subnucleus} \end{array}}. \end{array}$$

### 2.6. The Detection of Rat Facial Nerve Evoked Potential

The bridging spot of the facial nerve trunk was exposed before retrograde labeling of the facial nerve neurons. The nerve truck close to the heart end was stimulated at the bridging site. The recording electrode was inserted into the frontal muscle, and the grounding electrode was inserted into the ectogluteus. The stimulation current was applied every five minutes to help nerve and muscle recovery, and the stimulation was repeated three to four times. The stimulating current gradually increased until the maximum potential was induced and became stable. The evoked potential with the largest amplitude was the maximum evoked potential.

## 3. Statistical Analysis

All experimental data were analyzed with SPSS 13.0 statistical software. Before statistical analysis, *F* tests were performed on each dataset. A one-way analysis of variance (ANOVA) test or a Student *t*-test was used for between-group comparisons for data that conformed to the homogeneity of variance condition. The nonparametric test and the Kruskal-Wallis method were used for data that did not conform to the homogeneity of variance conditions. The Nemenyi test was used for each two-group comparison if differences were found. Differences were considered significant if *P* < 0.05.

## 4. Results

### 4.1. The General Morphological Observation of the Bridging Site of the Facial Nerve Trunk

The nerve conduit was not completely degraded nine weeks after surgery. Residual surgical sutures were still visible on the surface. When the bridge nerve conduit was cut open longitudinally, no apparent boundaries were observed between the newborn nerve end and the original nerve end. The trunk of the newborn nerve appeared ruddy. The surface of the newborn nerve trunk was smooth. No evident adhesion of the surrounding tissues or a suture reaction was observed. The regenerated nerve trunk inside the nerve conduit filled with only water was relatively thin for the control group with the nerve conduit.

**Figure 1:**
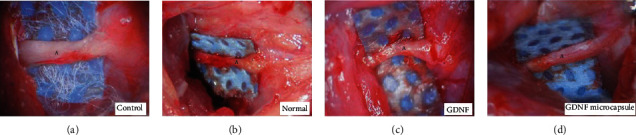
The general morphology of the bridging spot of the facial nerve trunk (9 weeks after surgery). (a) Normal facial nerve (uncut). In all three experimental groups, the regenerated nerve had already connected to the end of the nerve cut. A: the bridging spot of the facial nerve; (b) the nerve conduit filled with water; (c) the nerve conduit filled with GDNF solution; (d) the nerve conduit filled with GDNF microcapsules.

The control group had a thinner morphology and a rougher surface than test groups with nerve conduits filled with the GDNF or GDNF microcapsules. For the test group with the nerve conduit filled with GDNF solution, the morphology of the regenerated nerve trunk, with a smooth surface, was improved compared to that of the control group. The regenerated nerve trunk was still relatively thin but uniform. The newborn facial nerve trunk of the test group with nerve conduits filled with GDNF microcapsules was the most uniform in thickness. The surface was smooth with the best morphology, having the most resemblance to the regular facial nerve trunk (Figure 1).

### 4.2. The Analysis of the Number of the Facial Nerve Zygomatic and Buccal Branch within the Facial Nerve Nucleus

**Figure 2:**
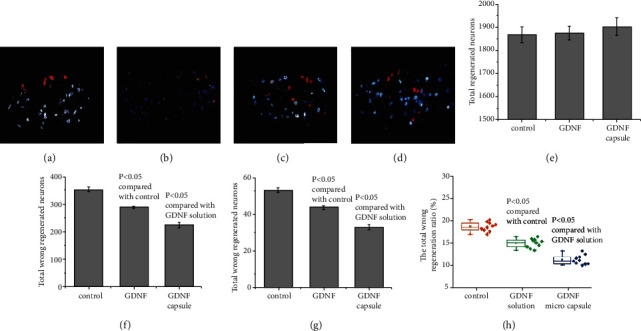
Distribution of motor neurons from the zygomatic branch and the buccal branch at the facial nerve nucleus. The blue color shows motor neurons of the buccal branch of the facial nerve stained with FG. The red color shows DIL-stained zygomatic motor neurons of the facial nerve. The claret color demonstrates the double-labeled neurons. (a) Normal distribution of motor neurons from the zygomatic branch and the buccal branch motor neurons; (b) normal control group (nerve conduit filled with water); (c) testing group with the nerve conduit filled with GDNF solution; (d) testing group with the nerve conduit filled with GDNF microcapsule; (e) summary of total regenerated neurons. Total regenerated neurons = total regenerated neurons in the zygomatic branch + total regenerated neurons in the buccal branch–double − labeled neurons; (f) summary of total incorrectly regenerated neurons. Total incorrectly regenerated neurons = incorrectly regenerated neurons in the zygomatic branch + incorrectly regenerated neurons in the buccal branch + double − labeled neurons; (g) summary of double-labeled neurons; (h) summary of the total incorrect regeneration ratio. Total incorrect regeneration ratio = total incorrectly regenerated neurons/total regenerated neurons in the zygomatic branch and buccal branches.

The distribution of motor neurons of the zygomatic and buccal branches at the facial nerve is shown in Figure 2.  Figure 2(a) shows the location of neurons of the zygomatic and buccal branches of the facial nerve in normal rats. Different amounts of double-labeled purple or claret-colored neurons are shown in Figure 2(b), suggesting that the regenerated nerve fibers came from the zygomatic and buccal branches' dual innervations. A similar phenomenon was observed in the testing groups with nerve conduits filled with either GDNF solution (Figure 2(c)) or GDNF microcapsules (Figure 2(d)). However, the total number of double-labeled neurons (Figure 2(g)) and the number of incorrectly regenerated facial nerve neurons (Figure 2(f)) from these two groups are fewer than those of the control group.

For the testing group with the nerve conduit filled with GDNF microcapsules, the total number of incorrectly regenerated facial nerve neurons and double-labeled neurons is fewer than that of the GDNF solution group. The difference is statistically significant (*P* < 0.05) (Figures 2(f)–2(h)). On the other hand, we also noticed that compared to that of the control testing group, the GDNF had little effect on total regenerated neurons (Figure 2(e)) (*P* > 0.05).

### 4.3. The Analysis of the Facial Nerve Evoked Potentials

**Figure 3:**
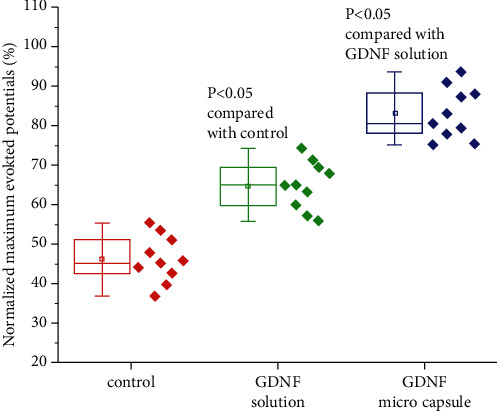
Functional analysis of the facial nerves regenerated in rats. The normalized maximum evoked potential is defined by the percentage of the maximum evoked potential recorded from the surgery side to the maximum evoked potential from the normal side of the same animal.

An electrophysiological recording is highly influenced by several external factors, such as AC power, environmental vibration, and status of the animal's anesthesia. Variation between different animals is significant; therefore, it is difficult to compare the absolute evoked potentials between animals. Consequently, we recorded the maximum evoked potentials from the bridged facial nerve side and the healthy facial nerve side of the same animal. We then normalized the evoked potentials of the bridged side of the facial nerve to the healthy side recording by calculating the ratio of the maximum evoked potential on the bridged side to that of the healthy side. The maximum evoked potentials of the three testing groups are summarized in Figure 3. The maximum facial nerve potentials evoked on the bridged side from the three testing groups are lower than those on the healthy side recording, suggesting that the facial nerve function from all three testing groups was not completely recovered. However, the maximum evoked potentials of the testing groups with the nerve conduit filled with GDNF solution or GDNF microcapsule were much higher than those of the control group (*P* < 0.05). In Figure 3, we observe that the maximum evoked potential of the GDNF microcapsule group is much higher than that of the nerve conduit filled with GDNF solution (*P* < 0.05). This finding suggests that the stable slow-released GNDF microcapsule inside the biodegradable nerve conduit can help to recover the function of the newborn regenerated nerve fibers.

## 5. Discussion

Some researchers have directly injected NTF into the silicone conduit to bridge damaged nerve fibers, but the results have been disappointing [12]. Since the injected NTFs may leak from the open ends of the nerve conduit and the silicone tube wall also prevents the surrounding nutrients from reaching the regenerated neuron axons, the actual dose of the NFTs around the regenerated neuron axons inside of the nerve conduit microenvironment may be pretty low. Other researchers also reported the local injection of NTFs, such as in the animal skin hypodermis, muscle, or abdomen. These local injections have the disadvantage of letting NTFs into the entire body's bloodstream and causing severe adverse reactions. As a result, researchers have tried to soak transplant materials in the solution containing NTFs and apply them locally [13]. Although the local concentration of NTFs increased, the available doses of NTFs and the duration are still minimal. With the rapid progress of biomaterial technology, researchers have tried to combine NTFs and nerve conduits to make nerve-composite conduits, such as polymer nerve conduits containing NTFs. Lizarraga-Valderrama and Ronchi had tried to add or implant Schwann cells, previously transfected with neurotrophic factor plasmids, into nerve conduits to achieve a regeneration microenvironment that could continuously release NTF locally [14]. The application of these methods significantly improved the effects of NFTs on nerve regeneration. However, due to technical limitations, high cost, the difficulty of large-scale preparation, and side effects, the actual application of these methods is highly restricted [13]. The other effort was to mix NTF with biological collagen to create a three-dimensional scaffold [15]. The three-dimensional scaffold is of a semicoagulated biodegradable material. The scaffold-supporting intensity can support axons that extend forward. At the same time, it can also leave enough space for axons to multiply.

Furthermore, the interspace can hold a certain number of NTFs and prevent the loss of NTFs. The NTFs inside the three-dimensional scaffold can be affected for 16 days [16]. This method is simple, low-cost, and easy to popularize. However, the NTF release time is still too short and the local concentration of NTFs is uneven. There is a sudden release effect of NTFs with this method.

Nowadays, the more mature and feasible method is the microcapsule technique. Compared with nanotechnology, its technical requirement is simple, the cost is relatively low, and the slow-release time is long enough. In this study, we added NTFs to microcapsules and then mixed them with biological collagen to make a three-dimensional scaffold. This technique provides a collagen-based three-dimensional scaffold and extends the release time to maintain a constant concentration of NTFs around the injured nerve. Theoretically, the growth rate of damaged neuron axons is about 1 to 2 mm a day [17]. The NTF microcapsules created in this study can be stably and continuously released for more than 40 days. The time is long enough for the regenerated neuron axons to pass through the bridging segments to distal endoneurial tubes. Our results indicate that the effect of slow-released GDNF microcapsules on neuron regeneration is much better than that of GDNF alone.

Our exciting findings are that the slow, continuously-released GDNF microcapsule within the nerve conduit cannot substantially increase the regenerated neuron numbers. Still, it strongly affects the guiding of axonal nerve regeneration. A previous study has reported that exogenous BDNF and GDNFs promote axon sprouting but do not increase the number of neurons that regenerate axons after nerve transection and surgical repair [18]. Reducing the amount of time spent on the nerve throughout the injury can reduce the wrong nerve regeneration, known as the acceleration theory [19]. Local nerves and Schwann cells within the myelin sheath can increase the production capacity in responding to peripheral nerve injuries [18]. However, the upregulation process is slow and the endogenous NTFs often cannot meet the needs for nerve regeneration. The application of exogenous NTF protects neurons and reduces apoptosis and shortens their injury time by enhancing the growth of the neural bud growth cone. This will help the injured nerve find the distal nerve end and reach the right distal end of the neural tube. Thus, the application of exogenous NTFs can guide the growth of the axon and reduce the wrong regeneration of nerve fibers.

Gordon described that after immediate repair of injured peripheral nerves with exogenous administration of NTFs, the number of neurons that regenerated their axons did not increase significantly. Regenerated axons in distal nerve stumps had access to NTFs expressed within the stumps [18]. These are retrogradely transported to the neuronal cell bodies and increase the expression of NTFs in the soma of axotomized neurons to support axonal regeneration. During these processes, the duration of the existence is the key factor. For example, motor neurons regenerated their axons when BDNF and/or GDNF were continuously infused at the suture site over the 28 days of axon regeneration [18]. In our study, the BDNF microcapsule in the nerve conduit ensured long, stable exposure of injured neurons to NTFs. Our results demonstrated an essential phenomenon that slow and steady continuous release of NTFs by microcapsules in the nerve conduit enhances the regeneration of neuron axons and plays a significant role in restoring nerve function.

This study chose a biodegradable synthetic material, PLGA/chitosan, to fabricate the nerve conduit. The nerve conduit provides a suitable carrier for the steady release of active GDNF microcapsules and offers an excellent microenvironment for facilitating nerve-guided regeneration. Furthermore, the technique has other advantages: (1) the manufacturing process of these two types of widely used materials is technically mature and convenient to customize and commercialize [20]; (2) the PLGA tube has good biological compatibility and biodegradable properties. Inside the body, it can maintain adequate physical support and avoid oppressing and even blocking nerve growth due to swelling or deformation. A good nerve repair material, chitosan, can be degraded to monosaccharides and has excellent biocompatibility. Chitosan can also reduce scar formation by inhibiting fibroblast growth [21]. Although PLGA degradation can produce a small amount of acid, causing damage to the nerve, the metabolite of chitosan is alkaline. This may neutralize the degradation products and partially offset the side effects of PLGA [14, 17]; (3) the PLGA/chitosan nerve conduit has a very stable degradation time. Both *in vitro* and *in vivo* experiments proved that the conduit could be degraded by itself in 1 to 3 months [22]. The process could avoid the entrapment of regenerated newborn nerves; and (4) such a type of biological conduit has excellent permeability and, therefore, can facilitate the exchanges of nutrients from the surrounding environment.

## 6. Conclusion

Bridging dissected rat facial nerve trunks by filling biodegradable nerve conduits with GDNF microcapsules can reduce the chance of incorrect nerve regeneration. The bridging effect is much better than the bridging by direct injection of the GDNF into the nerve conduit or bridging the nerve conduit without using any NTFs. The exciting results of this study provide an experimental foundation for the future treatment of facial nerve functional recovery and the translational application and clinical practice of this technique.

## Data Availability

All data could be found in the manuscript and previous literatures in the reference.
